# The Impact of Sustainable Development Technology on a Small Economy—The Case of Energy-Saving Technology

**DOI:** 10.3390/ijerph15020295

**Published:** 2018-02-08

**Authors:** Xiding Chen, Qinghua Huang, Weilun Huang, Xue Li

**Affiliations:** 1 School of Finance, Wenzhou Business College, Wenzhou 325035, Zhejiang, China; chanxd@wzbc.edu.cn; 2 College of Economics & Management, Southwest University, Chongqing 400715, China; hqh@swu.edu.cn; 3 School of International Economics and Trade, Shanghai Lixin University of Accounting and Finance, Shanghai 201620, China; lixue@lixin.edu.cn

**Keywords:** sustainable development technology, energy saving technology, macroeconomic variables in a small economy, stochastic analysis

## Abstract

We investigated the impact of a sustainable development technology on the macroeconomic variables in a small economy utilizing a case study with a stochastically improving energy saving technology and a stochastically increasing energy price. The results show the technological displacement effects of energy saving technology are stronger, but there are more ambiguous instantaneous returns to physical capital. However, the energy saving technology’s displacement effects might not affect the conditions under which the Harberger-Laursen-Metzler (HLM) effect holds. The effects of rising energy prices on bonds are stronger, and there are more ambiguous instantaneous returns, but the conditions under which the HLM effect holds are different.

## 1. Introduction

According to the literature on sustainable development technology, the specific future direction of energy technology will be more energy-saving due to sustainable energy policies and regulations from various governments, the price of energy rising relative to those of other goods, and research and development (R&D) efforts. The International Energy Agency [1] reported a reduction in global energy intensity (energy share of Gross Domestic Product, GDP) of 0.5% per year over the last decade and predicts a 1.8% per year decline by 2035. Narayanan and Sahu [2] found that the aggregate energy intensity of manufacturing industries decreased from 1990 to 2008. Hooker [3] demonstrated that energy intensity declined (implying energy-saving activities) in the U.S. from 1949 to 1999, and the intensity was 1.0% in the first quarter of 2000.

The sustainable energy policies and regulations implemented by governments have affected the energy-saving technologies available on the market. Many governments have used several types of policy tools to fund basic R&D, to support product launches, to reduce and overcome barriers to entry, and to provide suppliers and users the correct incentives to encourage them to employ energy-saving technologies. The policy tools include subsidizing R&D, technology and performance standards, subsidizing the distribution of energy-saving technology, taxes, and cap-and-trade systems, voluntary agreements, or a combination of such policies [4]. Jaffe et al. [5] reviewed U.S.A. federal climate technology initiatives (based on the 2004 budget) and found that energy conservation accounts for 41% of R&D spending, which is valued at approximately $1.3 billion a year, and 34% of adoption spending ($1.0 billion a year) goes to state energy efficiency grants. The International Energy Agency [1] proposed policies to increase energy efficiency that include increasing the visibility and affordability of energy efficiency, prioritizing energy efficiency, and making energy efficiency more practical and more mainstream.

As the price of energy rises relative to those of other goods, the production function or capital goods will contain more energy-saving technologies. The International Energy Agency [1] predicted that electricity prices will increase at an annual rate of 15% (by 2035) due to higher fuel prices, increased use of renewables, CO_2_ pricing, etc. Firms will use labor to produce when energy price is cheaper and to replace their capital goods (such as cars) with more energy-efficient models that are available on the market [6].

R&D effort is a key element in developing energy-saving technology and has the potential to drive down the costs for firms. The International Energy Agency [7] uses experience curves and the progress ratios of the power-generating sector to understand why new technology has reduced costs over time. The progress ratio is the degree to which the cost of installing a technology reduces when the total amount of installed energy technology doubles. The progress ratio for photovoltaic modules in the world market in the period of 1976–1992 was 82% (a price reduction by 18%); the figure for Danish wind turbines in the period of 1982–1997 was 96%; and the value in the wind power sector in the United States (European Union) in the period of 1985–1994 (1980–1995) was 68% (82%).

The energy-saving technology should be a joint process that can be affected by various means of energy generation (like coal, gas, nuclear, and renewables), market experience, government policies, the development path of technology, etc. As the International Energy Agency [1] stated, the electricity demand in emerging economies (like China and India) drives a 70% increase of worldwide demand with renewables accounting for half of its new capacity. The International Energy Agency [4] suggests that technical change is a cyclical process that is affected by the interaction of market experience and further technological development. Hooker [3] showed that U.S. energy intensity declined fairly steadily during the period of 1949–2000, with several small rises but more rapid declines. The International Energy Agency [7] termed an R&D breakthrough in production as structural technology change that results in a radical change in technology, such as new temperature-resistant materials for gas turbines. Roehrl and Riahi [8] used a cost assessment of greenhouse gas emissions mitigation efforts to study technological dynamics and found that technological improvement develops in a “path-dependent” direction, where the cost of changing the course of this development increases after a particular initial point.

There are many economic studies on sustainable development technologies, but few have discussed the impact of energy-saving technologies on macroeconomic variables. To fill this research gap, we investigated the impact of an increasing variety of energy-saving technologies and energy prices on the growth rate of real wealth, and the Harberger-Laursen-Metzler (HLM) effect of a small open economy. According to Arrow [9], Kamien and Schwartz [10], Reinganum [11], and Clarke and Weyant [12], the development of additional energy-saving technologies would be the endogenous equilibrium of the economic system, which could be affected by policy. However, such technologies may receive a sub-optimal level of investment from the private sector. As argued by Romer [13,14], Grossman and Helpman [15], Aghion and Howitt [16], Clarke and Weyant [12], and Acemoglu [17], the spillovers from energy-saving technologies might be a source of steady, long-term economic growth. The International Energy Agency [1] estimated that energy efficiency and more energy-saving technologies could improve energy security and economic growth and could increase the total global economic output through 2035 by $18 trillion.

For a small economy, the literature has demonstrated that the validity or invalidity of the HLM effect depends on the economic factors or the specific formulations of the model. In this paper, they are energy-saving technologies, an increasing price of energy, and their cross-variance. This paper focuses on the impact of energy price and its association with physical capital and bonds. The HLM effect is the specific effect of terms of trade (TOT) shocks on the economy, meaning that, when the levels of investment and income are fixed, a deterioration in the TOT will raise real expenditures, reduce savings, and cause a deterioration in the current account [18,19]. Many research studies have explained how energy price fluctuations can affect macroeconomic performance [20]. The energy price (increasing and stochastic) can have effects on the labor market, aggregation and allocation, interest rates, and petroleum product market channels [21,22]. Hamilton [23] further argued that increases in energy prices might be a factor in U.S. macroeconomic performance. Davis and Haltiwanger [21] demonstrated the asymmetric effects of oil price shocks on the creation and destruction of U.S.A. manufacturing jobs.

As the time path of energy-saving technology should be a stochastically increasing process, this paper shows that the uncertainty of energy-saving technology can impact macroeconomic variables. We employed a continuous-time, representative household, stochastic optimizing model to examine and evaluate the impact of an increasing variety of energy-saving technologies and energy prices on the growth rate of real wealth, and the Harberger–Laursen–Metzler (HLM) effect of a small open economy. Many researchers have used deterministic models to discuss the economic factors of the HLM effect. Obstfeld [24] and Svesson and Razin [25] used a household intertemporal utility optimization model to show how the HLM effect would fail to hold when time preferences are increasing in utility. Persson and Svensson [26] and Mansoorian [27] constructed a habit persistence and infinite horizon model to demonstrate that the HLM effect holds if the marginal utility of consumption is increasing. Ikeda [28] used a model with weakly non-separable preferences to find that the HLM effect occurs if consumers’ preferences toward imports increase in wealth.

We analyzed the effects of changes in energy-saving technology and energy prices on the consumption, saving, and wealth of a country. We provide a theoretical model where individuals decide how much to consume (domestic and foreign goods) and how much to save (investments in capital or bonds). Domestic production levels are determined by the input usages (labor, capital, and energy) and production technology. We assume that the changes in energy-saving technology, energy prices, and the prices of foreign goods are exogenous and focuses on how the unanticipated shocks in energy-saving technology and energy prices affect the wealth of a country as well as the levels of consumption and saving.

The rest of this paper is organized as follows. Section 2 develops the analytical framework that the impact of energy-saving technology on a small economy. Section 3 analyses macroeconomic variables of a small economy by energy-saving technologies’ impact. The last section offers conclusions.

## 2. The Analytical Framework

We assume a small open economy with exogenous prices of foreign goods and interest rate, two perfectly competitive and complete markets (a commodity and a financial asset), a representative household (no firms), no externalities or trade barriers (no tariffs or freight costs), and three goods (energy and a final good in a foreign country that is imported from abroad and a final good in the home country). A representative household uses energy, labor, and capital to produce the final good and consumes the final good and the imported good. The context is set by paradigmatic articles related to the analytical framework, such as those by Sadorsky [29] and Turnovsky [30,31].

The path of the energy price should be determined through a process of stochastically increasing prices of oil and other energy products, and the mean and volatility of the energy price should affect the macroeconomic variables. Thus, the relative price of energy ($P_{e}$, the price of the final good in the home country is fixed at 1) is exogenously given and generated by the Brownian motion process, using the following formula:

(1) $${\mathrm{dP}}_{e}/P_{e}{=\alpha +\mathrm{du}}_{e},$$

where α is the instantaneous expected rate of change in the relative energy price, and ${\mathrm{du}}_{e}$ is a random variable with mean zero and variance, $\sigma_{e}^{2}$dt. Sadorsky [29] used a vector autoregression to demonstrate that oil prices and oil price volatility can affect economic activity (stock returns).

The relative price of the final good in foreign country ($P_{f}$) is

(2) $${\mathrm{dP}}_{f}/P_{f}{=\mathrm{du}}_{f},$$

where ${\mathrm{du}}_{f}$ is a random variable with mean zero and variance $\sigma_{f}^{2}$dt. The relative price of the imported goods ($P_{i}$) is $P_{i}{=P}_{e}^{\theta_{1}}P_{f}^{{1-\theta }_{1}}$, which is aggregated by the prices of energy and the final good in a foreign country. $\theta_{1}$ is the ratio of the imported value of energy by the total imported value, so $0<\theta_{1}<1$. From Equations (1) and (2), TOT should be $1/P_{i}$ and follows the Brownian motion process:

(3) $${\mathrm{dP}}_{i}/P_{i}=\beta \mathrm{dt}+\theta_{1}{\mathrm{du}}_{e}{+(1-\theta }_{1}){\mathrm{du}}_{f},$$

where ${\beta =\alpha \theta }_{1}{-\theta }_{1}{(1-\theta }_{1}{)(\sigma }_{e}^{2}{+\sigma }_{f}^{2})/2$, which is the instantaneous expected change rate of TOT, is different from the assumption of Turnovsky [30,31], which considers only one imported good.

Final good in home country ($Y_{h}$) is produced by the inputs of labor (L), capital (K), and energy (E), which are measured by the final good in the home country. Let L = 1 and physical capital is the summary of capital and the imported value of energy, so its production function is

(4) $$Y_{h}{=T}_{e}{f(T}_{l}{,K,P}_{e}{E)=T}_{e}T_{l}^{\theta_{2}}{{(K+P}_{e}E)}^{{1-\theta }_{2}},$$

where $T_{e}$ is energy-saving technical displacement; $T_{l}$ is the effective labor force, which could be seen as the labor augmenting technical change; $P_{e}E$ is the inputted (read: imported) value of energy; $0<\theta_{2}<1$. (Labor, capital, and energy are the necessity to produce the final good in home country, so ${f(\;0,\;K,\;P}_{e}{E)=f(T}_{l}{,\;0,\;P}_{e}{E)=f(T}_{l},\;K,\;0)=0$).

$T_{e}$ and $T_{l}$ are exogenous variables, so ${\mathrm{dT}}_{e}/T_{e}{=\delta \mathrm{dt}+\mathrm{du}}_{T_{e}}$ and ${\mathrm{dT}}_{l}/T_{l}{=\mathrm{du}}_{T_{l}}$ are assumed, where ${\mathrm{du}}_{T_{e}}$ and ${\mathrm{du}}_{T_{l}}$ are random variables with mean zero and variance $\sigma_{T_{e}}^{2}$dt and $\sigma_{T_{l}}^{2}$dt. Based on the existing literature, more and more energy-saving technologies should be contained for physical capital and energy, so $T_{e}$ might be closer to the total factor productivity in conventional settings.

From Equations (1) and (4) about energy-saving technical displacement and effective labor force, the stochastic production function is

(5) $${\mathrm{dY}}_{h}{=Y}_{h}{\{[1+\delta -\theta }_{2}{(1-\theta }_{2}{)(\sigma }_{T_{l}}^{2}{+E}_{R}^{2}\sigma_{e}^{2})/{2]\mathrm{dt}+\mathrm{du}}_{T_{e}}{+\theta }_{2}{\mathrm{du}}_{T_{l}}{+(1-\theta }_{2}{)E}_{R}{\mathrm{du}}_{e}\},$$

where $E_{R}{=P}_{e}E/{(K+P}_{e}E)>0$ is constant.

The model is real (no money), and the representative household holds two securities (traded bonds and claims on capital) that generate returns as stipulated by an exogenous interest rate (r), such that the rate of return on physical capital (${\mathrm{MP}}_{{K+P}_{e}E}$) is equal to the interest rate necessary to satisfy the long-term equilibrium condition of the financial asset market, which is

(6) $${\mathrm{MP}}_{{K+P}_{e}E}{=(1-\theta }_{2}{)Y}_{h}/{(K+P}_{e}E)=r,$$

The instantaneous utility of the representative household is decided by the consumption of final good in home country and in foreign country ($C_{h}$dt, $C_{f}$dt), which can be written as $U\left(C_{h}{,C}_{f}\right){=(C}_{h}^{\theta_{3}}C_{f}^{{1-\theta }_{3}}{)}^{\rho_{1}}/\rho_{1}$, where $-\infty <\rho_{1}<1$, and $0<\theta_{3}<1$. The value function of the representative household at t ($V_{t}\left(C_{h}{,C}_{f}\right)$) is its expected value of discounted utility at t, which is

(7) $$V_{t}\left(C_{h}{,C}_{f}\right){=\mathrm{Max}E}_{t}\int_{t}^{\infty }{{(C}_{h}^{\theta_{3}}C_{f}^{{1-\theta }_{3}})}^{\rho_{1}}/\rho_{1}e^{{-\rho }_{2}S}\mathrm{ds}$$

where $\rho_{2}$ is time preference rate of the representative household.

The representative household’s real wealth (W) is summary of capital and the relative value of bond ($P_{i}B$, which is valued by the relative price of the imported goods), so the wealth is given by ${W=K+P}_{i}B$. Let $N_{K}=K/W>0$ and $N_{B}{=P}_{i}B/W$ be the shares of capital and bond in portfolio held of the representative household. Then, the portfolio shares adding up condition is

(8) $$N_{K}{+N}_{B}=1.$$

The instantaneous returns of physical capital (capital and energy) and bond (${\mathrm{dR}}_{{K+P}_{e}E}$, ${\mathrm{dR}}_{B}$), the instantaneous consumption values of final good in home country and in foreign country ($C_{h}$dt, $P_{i}C_{f}$dt), and instantaneous production input of energy ($P_{e}\mathrm{Edt}$) are the sources of wealth accumulation. For $E_{R}$ is constant, the share of energy in portfolio held by the representative household is $N_{P_{e}E}{=P}_{e}E/W=E_{R}N_{K}/{(1-E}_{R})$.

For Equations (5) and (6), ${\mathrm{dR}}_{{K+P}_{e}E}{=\mathrm{dY}}_{h}/{(K+P}_{e}E)$ and Let ${d(K+P}_{e}E)=0$, the instantaneous returns of physical capital is

(9) $${\mathrm{dR}}_{{K+P}_{e}E}=r\{[1+\delta -\theta_{2}(1-\theta_{2})(\sigma_{T_{l}}^{2}+E_{R}^{2}\sigma_{e}^{2})/2]\mathrm{dt}+{\mathrm{du}}_{T_{e}}{+\theta }_{2}{\mathrm{du}}_{T_{l}}{+(1-\theta }_{2}{)E}_{R}{\mathrm{du}}_{e}\}/{(1-\theta }_{2})$$

For Equation (3) and ${\mathrm{dR}}_{B}{=[\mathrm{rP}}_{i}{\mathrm{Bdt}+d(P}_{i}B)]/P_{i}B$, the instantaneous returns of bond is

(10) $${\mathrm{dR}}_{B}=(r+\beta )\mathrm{dt}+\theta_{1}{\mathrm{du}}_{e}{+(1-\theta }_{1}{)\mathrm{du}}_{f}.$$

For Equations (9) and (10), the stochastic wealth accumulate equation is

(11) $${\mathrm{dW}=W[(N}_{K}{+N}_{P_{e}E}{)\mathrm{dR}}_{{K+P}_{e}E}{+N}_{B}{\mathrm{dR}}_{B}{-(N}_{C}{+N}_{P_{e}E})\mathrm{dt}]\;{=W\{[\phi N}_{K}{+(r+\beta )N}_{B}{-N}_{C}{]\mathrm{dt}+\mathrm{rN}}_{K}{(\mathrm{du}}_{T_{e}}{+\theta }_{2}{\mathrm{du}}_{T_{l}})/{[(1-E}_{R}{)(1-\theta }_{2})]\;{+(1-\theta }_{1}{)N}_{B}{\mathrm{du}}_{f}{+[\mathrm{rE}}_{R}N_{K}/{(1-E}_{R}{)+\theta }_{1}N_{B}{]\mathrm{du}}_{e}\}$$

where ${\phi =\{r\{[1+\delta -\theta_{2}(1-\theta_{2})(\sigma_{T_{l}}^{2}+E_{R}^{2}\sigma_{e}^{2})/2]-E}_{R}{(1-\theta }_{2})\}/{[(1-\theta }_{2}{)(1-E}_{R})]$, and $N_{C}{=(C}_{h}{+P}_{f}C_{f})/W$ is the share of consumption in portfolio held of the representative household.

The optimization is to choose $C_{h}$, $C_{f}$, $N_{K}$, and $N_{B}$ to maximize Equation (7) subject to Equations (2), (3), (8), and (11) and follow the procedure set out in Turnovsky [30,31,32]. Define aggregate consumption C by $C\equiv C_{h}{+P}_{f}C_{f}$. The first-order optimality conditions are

(12a) $$C_{h}{=\theta }_{3}C$$

(12b) $$C_{f}{=(1-\theta }_{3})C$$

(12c) $$N_{C}{=\{\rho }_{2}{-\rho }_{1}{[\phi N}_{K}{+(r+\beta )N}_{B}{]+\beta \rho }_{1}{(1-\theta }_{3})\}/{(1-\rho }_{1})\;{-\rho }_{1}{\{\{\rho }_{1}{(1-\theta }_{3}{)N}_{B}{+(1-\theta }_{3}{)[\rho }_{1}{(1-\theta }_{3})-1]/{2(1-\rho }_{1}{)\}[\theta }_{1}^{2}\sigma_{e}^{2}{+(1-\theta }_{1}{)}^{2}\sigma_{f}^{2}]/{(1-\rho }_{1})\;{+r}^{2}N_{K}^{2}{(\sigma }_{T_{e}}^{2}{+\theta }_{2}^{2}\sigma_{T_{l}}^{2})/{{[(1-\theta }_{2}{)(1-E}_{R})]}^{2}{+(1-\theta }_{1}{)}^{2}N_{B}^{2}\sigma_{f}^{2}{+[\mathrm{rE}}_{R}N_{K}/{(1-E}_{R}{)+\theta }_{1}N_{B}{]}^{2}\sigma_{e}^{2}\}/2$$

(12d) $${\phi -(1-\rho }_{1}{)N}_{K}{\{r}^{2}{(\sigma }_{T_{e}}^{2}{+\theta }_{2}^{2}\sigma_{T_{l}}^{2})/{{[(1-\theta }_{2}{)(1-E}_{R})]}^{2}+{\mathrm{rE}}_{R}{[\mathrm{rE}}_{R}N_{K}/{(1-E}_{R}{)+\theta }_{1}N_{B}{]\sigma }_{e}^{2}/{(1-E}_{R})\;{=r+\beta +(1-\rho }_{1}{)\{\theta }_{1}{[\mathrm{rE}}_{R}N_{K}/{(1-E}_{R}{)+\theta }_{1}N_{B}{]\sigma }_{e}^{2}{+(1-\theta }_{1}{)}^{2}N_{B}\sigma_{f}^{2}{-\rho }_{1}^{2}{(1-\theta }_{3}{)[\theta }_{1}^{2}\sigma_{e}^{2}{+(1-\theta }_{1}{)}^{2}\sigma_{f}^{2}]\}.$$

Equations (12a) and (12b) describe the consumption of the final good in the home country and in the foreign country as fixed shares of overall consumption expenditures. The mean of Equation (12c) is the aggregate consumption–wealth ratio, which depends on portfolio share and preference parameters, permanent and temporary shocks, and the value and production functions of the representative household.

Solving Equations (8) and (12d) for the equilibrium portfolio shares of physical capital and bond, we have $N_{B}$ as

(13) $$N_{B}=(r+\beta -\phi )/R_{a}{+\{(1-\rho }_{1}{)[r}^{2}{(\sigma }_{T_{e}}^{2}{+\theta }_{2}^{2}\sigma_{T_{l}}^{2})/{{[(1-\theta }_{2}{)(1-E}_{R})]}^{2}{+\mathrm{rE}}_{R}{[\mathrm{rE}}_{R}/{(1-E}_{R}{)-\theta }_{1}{]\sigma }_{e}^{2}/{(1-E}_{R})]\;{-\rho }_{1}{(1-\theta }_{3}{)[\theta }_{1}^{2}\sigma_{e}^{2}{+(1-\theta }_{1}{)}^{2}\sigma_{f}^{2}{]\}/R}_{a}$$

where $R_{a}{=(1-\rho }_{1}{)\{r}^{2}{(\sigma }_{T_{e}}^{2}{+\theta }_{2}^{2}\sigma_{T_{l}}^{2})/{{[(1-\theta }_{2}{)(1-E}_{R})]}^{2}{+[\mathrm{rE}}_{R}/{(1-E}_{R}{)-\theta }_{1}{]}^{2}\sigma_{e}^{2}{+(1-\theta }_{1}{)}^{2}\sigma_{f}^{2}\}$ is the risk adjudge factor of $N_{B}$. Equation (13) could indicate the representative household’s speculative and hedging behavior regarding bonds. The speculative component depends on the difference between the differential between the expected real return rates on bonds and physical capital, and the hedging component depends on the variances in the energy price, the final good in the foreign country, energy-saving technical displacement in energy production, and the effect of labor on technological improving.

For Equations (8), (12c) and (13), the equilibrium $N_{K}$, $N_{P_{e}E}$, $N_{B}$, $N_{C}$ should be constancy, then the growth rate of the representative household is

(14) $$\mathrm{dC}/{C=d(K+P}_{e}E)/{(K+P}_{e}{E)=\mathrm{dP}}_{i}B/P_{i}B=\mathrm{dW}/W\;{=[\phi N}_{K}{+(r+\beta )N}_{B}{-N}_{C}{]\mathrm{dt}+\mathrm{rN}}_{K}{(\mathrm{du}}_{T_{e}}{+\theta }_{2}{\mathrm{du}}_{T_{l}})/{[(1-E}_{R}{)(1-\theta }_{2})]\;{+(1-\theta }_{1}{)N}_{B}{\mathrm{du}}_{f}{+[\mathrm{rE}}_{R}N_{K}/{(1-E}_{R}{)+\theta }_{1}N_{B}{]\mathrm{du}}_{e}.$$

where the expected value should be equal to the difference between the earning rate and the shares of aggregated consumption of wealth.

From Equation (14), if the dynamic adjustments in the shares of capital, bonds, and consumption are not considered, the permanent energy-saving technical displacement (δ) will be larger, or the variance in the size of the effective labor force ($\sigma_{T_{l}}^{2}$) will be smaller, and the permanent growth rate of the economy will be faster. Moreover, the larger the permanent change in the energy price (α) or the lower the variance in the final good’s price in the foreign country ($\sigma_{f}^{2}$), the faster or slower the permanent growth rate of the energy price will be when the representative household is a net creditor ($N_{B}>0$) or debtor ($N_{B}<0$), respectively. However, the variance in energy-saving technical displacement ($\sigma_{T_{e}}^{2}$) might not change its permanent growth rate, and the effect of the energy price’s variance ($\sigma_{e}^{2}$) on its permanent growth rate will depend on the shares of capital and bonds held by the representative household. (The absolute value of $\left[{\phi N}_{{K+P}_{e}E}{+(r+\beta )N}_{B}{-N}_{C}\right]$ should be small enough to be consistent the transversality condition.)

## 3. Analysis of the Macroeconomic Variables

Many papers have proved the macroeconomic effects (growth rate and HLM effect) of permanent, temporary, anticipated, or unanticipated production. The production and TOT shocks are different [25,26,30,31,33,34]. Svensson and Razin [25] show that a temporary (permanent) TOT deterioration implies a deterioration (ambiguous effect) of the trade balance.

To analyze the permanent and temporary impact of energy-saving technology (the changes of δ and $\sigma_{T_{e}}^{2}$) and other shocks (the changes of α, $\sigma_{f}^{2}$, $\sigma_{T_{l}}^{2}$, and $\sigma_{e}^{2}$) on the growth rate and HLM effect of a small economy, Equation (13) is differentiated by the above shocks. The solutions are

(15) $$\partial N_{B}/\partial \delta <0,\partial N_{B}/\partial \sigma_{T_{e}}^{2}>0;\partial N_{B}/\partial \alpha >0,\partial N_{B}/\partial \sigma_{f}^{2}<0,\partial N_{B}/\partial \sigma_{T_{l}}^{2}>0,\partial N_{B}/\partial \sigma_{e}^{2}\begin{array}{c} >\\ =\\ < \end{array}0.$$

To illustrate an explicit mathematical derivation for proving the existence of more ambiguous instantaneous returns to physical capital and bonds due to the permanent technological displacement effects of energy saving technology, we found $\partial N_{B}/\partial \delta =-r/[\left({1-\theta }_{2}\right)\left({1-E}_{R}\right)R_{a}]<0,$ for Equation (8), $\partial N_{K}/\partial \delta =r/[\left({1-\theta }_{2}\right)\left({1-E}_{R}\right)R_{a}]>0$. Thus, an increase in δ would raise the expected return to physical capital, leading to an increasing share of physical capital being held by the rational representative household. Its impact factors for the increment on share of physical capital are exogenous interest rate (r), the share of physical capital in its production function ($1-\theta_{2}$), the share of capital in its physical capital ($1-E_{R}$), and the risk adjudge factor of $N_{B}$ ($R_{a}$). However, in Equation (8), the share of bonds is forced to decrease.

According to Equation (15), for the representative household, increases in $\sigma_{T_{e}}^{2}$ and $\sigma_{T_{l}}^{2}$ raise the level of uncertainty associated with physical capital, so, for a risk-averse representative household, given ($-\infty <\rho_{1}<1$) and Equation (8), its share of bonds must increase. An increase in α ($\sigma_{f}^{2}$) raises the expected return (uncertainty) on bonds, leading to an increasing (decreasing) share of bonds being held by a rational (risk-averse) representative household. An increase in $\sigma_{e}^{2}$ raises the uncertainty associated with physical capital and bonds. Therefore, whether such an increase results in an increasing, constant, or decreasing share of bonds depends on the parameters associated with the relative price of the imported good, the production function, and the value function. These effects are similar to the results obtained by Dreze and Modigliani [35] and other papers analyzing consumption, production, and investment decisions under uncertainty.

The solutions that Equation (12c) is differentiated by the above shocks are

(16) $$\begin{array}{c} \partial N_{C}/\partial \delta ,\partial N_{C}/\partial \sigma_{T_{e}}^{2},\partial N_{C}/\partial \alpha ,\\ \partial N_{C}/\partial \sigma_{f}^{2},\partial N_{C}/\partial \sigma_{T_{l}}^{2},\partial N_{B}/\partial \sigma_{e}^{2} \end{array}\begin{array}{c} >\\ =\\ < \end{array}0.$$

To illustrate the explicit mathematical derivation for proving the existence of more ambiguous instantaneous returns to the aggregate consumption–wealth ratio due to the permanent technological displacement effects of energy saving technology, we found

(17) $$\partial N_{C}/\partial \delta =-\left[\rho_{1}/\left(1-\rho_{1}\right)\right]{\{\mathrm{rN}}_{K}/[\left(1-\theta_{2}\right)\left(1-E_{R}\right)]+\left(\partial N_{K}/\partial \delta \right)[\phi -r-\beta \;-\rho_{1}{(1-\theta }_{3}{)[\theta }_{1}^{2}\sigma_{e}^{2}{+(1-\theta }_{1}{)}^{2}\sigma_{f}^{2}]/2\left(1-\rho_{1}\right)+r^{2}N_{K}{(1-\rho }_{1})(\sigma_{T_{e}}^{2}+\theta_{2}^{2}\sigma_{T_{l}}^{2})/{{[(1-\theta }_{2}{)(1-E}_{R})]}^{2}\;-{{(1-\theta }_{1})}^{2}{(1-\rho }_{1})\left(1-N_{K}\right)\sigma_{f}^{2}+{\mathrm{rN}}_{K}{(1-\rho }_{1}{)E}_{R}^{2}\sigma_{e}^{2}/{\left(1-E_{R}\right)}^{2}\;+{r\theta }_{1}E_{R}\sigma_{e}^{2}{(1-\rho }_{1})\left(1-2N_{K}\right)/{(1-E}_{R})\;-\theta_{1}^{2}\sigma_{e}^{2}{(1-\rho }_{1}{)(1-N}_{K})\}.$$

For the above formula, $\partial N_{C}/\partial \delta$ would be positive, zero, or negative, which depends on the relative values of all parameters.

According to Equation (16), an increase in δ ($\sigma_{T_{e}}^{2}$ and $\sigma_{T_{l}}^{2}$) would raise (reduce) the expected return of welfare and the output of the final good in the home country, an increase in α ($\sigma_{e}^{2}$) would raise (reduce) the expected welfare and the price of energy (the output of the final good in the home country), and an increase in $\sigma_{f}^{2}$ would reduce the expected return of welfare. However, due to the income and substitution effects, whether these changes would result in an increasing, constant, or decreasing share of consumption depends on all of the model parameters.

From Equation (11), the covariance of dW/W between ${\mathrm{du}}_{T_{e}}$, ${\mathrm{du}}_{T_{l}}$, ${\mathrm{du}}_{f}$, and ${\mathrm{du}}_{e}$ are

(18) $$\mathrm{cov}(\mathrm{dW}/{W,\mathrm{du}}_{T_{e}}{)=\{r\sigma }_{T_{e}}^{2}N_{K}/{[(1-E}_{R}{)(1-\theta }_{2})]\}\mathrm{dt}>0,\;\mathrm{cov}(\mathrm{dW}/{W,\mathrm{du}}_{T_{l}}{)=\{r\theta }_{2}N_{K}\sigma_{T_{l}}^{2}/{[(1-E}_{R}{)(1-\theta }_{2})]\}\mathrm{dt}>0,\;\mathrm{cov}(\mathrm{dW}/{W,\mathrm{du}}_{f}{)=(1-\theta }_{1}{)N}_{B}\sigma_{f}^{2}\mathrm{dt},\;\mathrm{cov}(\mathrm{dW}/{W,\mathrm{du}}_{e}{)=[\mathrm{rE}}_{R}N_{K}/{(1-E}_{R}{)+\theta }_{1}N_{B}{]\sigma }_{e}^{2}\mathrm{dt}.$$

According to Equation (18), the effects of the unanticipated productivity growth (${\mathrm{du}}_{T_{e}}$, ${\mathrm{du}}_{T_{l}}$) on the growth rate of the representative household are positive; thus, stochastic productivity growth would raise the expected return on physical capital, leading to an increasing rate of wealth accumulation. The effects of the unanticipated TOT deterioration (${\mathrm{du}}_{f}$, ${\mathrm{du}}_{e}$) on the growth rate of the representative household are determined by its share of bonds. When $N_{B}{<-rE}_{R}N_{K}/{[\theta }_{1}{(1-E}_{R})]$ (the representative household is a debtor), following a stochastic deterioration in TOT induced by the price of energy or the final good in the foreign country, the HLM effect holds. When $0>N_{B}>-rE_{R}N_{K}/{[\theta }_{1}{(1-E}_{R})]$ (debtor), following a stochastic deterioration in TOT induced by the price of the final good in the foreign country, the HLM effect holds, but a stochastic deterioration in TOT induced by the energy price would imply that the HLM effect does not hold. When $N_{B}>0$ (creditor), any stochastic deterioration in TOT would eliminate the HLM effect.

From Equation (11), the expected values of dW/W differentiated by the above shocks are

(19) $$\begin{array}{c} \begin{array}{c} \partial E(\mathrm{dW}/W)/\partial \delta ,\partial E(\mathrm{dW}/W)/\partial \alpha ,\\ \partial E(\mathrm{dW}/W)/\partial \sigma_{T_{e}}^{2},\partial E(\mathrm{dW}/W)/\partial \sigma_{T_{l}}^{2}, \end{array}\\ \begin{array}{c} \partial E(\mathrm{dW}/W)/\partial \sigma_{f}^{2},\partial E(\mathrm{dW}/W)/\partial \sigma_{e}^{2},\\ \partial \mathrm{var}(\mathrm{dW}/W)/\partial \sigma_{T_{e}}^{2},\partial \mathrm{var}(\mathrm{dW}/W)/\partial \sigma_{T_{l}}^{2},\\ \partial \mathrm{var}(\mathrm{dW}/W)/\partial \sigma_{f}^{2},\partial \mathrm{var}(\mathrm{dW}/W)/\partial \sigma_{e}^{2}, \end{array} \end{array}\begin{array}{c} >\\ =\\ < \end{array}\;0.$$

To illustrate an explicit mathematical derivation for proving the existence of more ambiguous instantaneous returns to the expect value of the wealth growth rate due to the permanent technological displacement effects of energy saving technology, we found

(20) $$\partial E(\mathrm{dW}/W)/\partial \delta =\left[(1-2\rho_{1})/\left(1-\rho_{1}\right)\right]{\{\mathrm{rN}}_{K}/[\left(1-\theta_{2}\right)\left(1-E_{R}\right)]+\left(\partial N_{K}/\partial \delta \right)\left(\phi -r-\beta \right)\}\;\left(\partial N_{K}/\partial \delta \right)[-\rho_{1}^{2}{(1-\theta }_{3}{)[\theta }_{1}^{2}\sigma_{e}^{2}{+(1-\theta }_{1}{)}^{2}\sigma_{f}^{2}]/2{\left(1-\rho_{1}\right)}^{2}\;-r^{2}N_{K}\rho_{1}(\sigma_{T_{e}}^{2}+\theta_{2}^{2}\sigma_{T_{l}}^{2})/{{[(1-\theta }_{2}{)(1-E}_{R})]}^{2}\;+{(1-\theta }_{1}{)}^{2}\rho_{1}\left(1-N_{K}\right)\sigma_{f}^{2}-{\mathrm{rN}}_{K}\rho_{1}E_{R}^{2}\sigma_{e}^{2}/{\left(1-E_{R}\right)}^{2}-{r\theta }_{1}E_{R}\sigma_{e}^{2}\rho_{1}\left(1-2N_{K}\right)/{(1-E}_{R})\;+\theta_{1}^{2}\sigma_{e}^{2}\rho_{1}{(1-N}_{K})\}.$$

For the above formula, ∂E(dW/W)/∂δ would be positive, zero, or negative which depends on the relative values of all parameters.

According to Equation (19), the effects of anticipated productivity growth (δ) and TOT deterioration (α), the increased variances in productivity ($\sigma_{T_{e}}^{2}$, $\sigma_{T_{l}}^{2}$) and TOT ($\sigma_{f}^{2}$, $\sigma_{e}^{2}$) on the wealth growth rate of the representative household, and the variances in their wealth growth rates are all determined by the share of bonds held by the household (debtor or creditor), the income and substitution effects, and the household’s degree of risk aversion.

Thus, the HLM effect of anticipated deterioration in TOT will hold under certain conditions. For example, the effects of increasing δ, α, $\sigma_{T_{e}}^{2}$, $\sigma_{T_{l}}^{2}$, $\sigma_{f}^{2}$, and $\sigma_{e}^{2}$ on their growth rates would be equal to the difference between the impacts of welfare and consumption. Increases in $\sigma_{T_{e}}^{2}$, $\sigma_{T_{l}}^{2}$, $\sigma_{f}^{2}$, and $\sigma_{e}^{2}$ would increase the variances in their growth rates when the shares of capital, bonds, and consumption are given. However, increasing $\sigma_{T_{e}}^{2}$ and $\sigma_{T_{l}}^{2}$ should reduce the share of capital, an increase in $\sigma_{f}^{2}$ should reduce the share of bonds, and an increase in $\sigma_{e}^{2}$ should reduce the shares of capital and bonds, in turn reducing the variances in their growth rates when the impacts of $\sigma_{T_{e}}^{2}$, $\sigma_{T_{l}}^{2}$, $\sigma_{T_{f}}^{2}$, and $\sigma_{e}^{2}$ on the variances in their growth rates are given. Thus, the results of Equation (18) could be positive, zero, or negative. This is verified by the fact that Kalulumia and Nyankiye [36] generated positive correlations between investment, saving, and the HLM effect. In addition, Bouakez and Kano [37] used data from Australia, Canada, and the United Kingdom to show that terms-of-trade movements do not affect the current account (no HLM effect).

## 4. Conclusions

This paper validates the impacts of sustainable development technology on the macroeconomic variables in a small economy. The identified variables hopefully can elicit a possible response for the government to make in establishing a policy to support green growth by sustainable development technology. At the same time, the enterprise who wants to penetrate into those countries in green growth can be more collaborative by understanding the possible impacts in their decision-making process.

Many literatures of sustainable development technology and sustainable energy policies have been suggested that the future direction of energy technology will be driven by energy-saving. Although studies on sustainable development technologies are numerous in engineering, very few have discussed the impact of energy-saving technologies by looking at the macroeconomic variables.

Here, a continuous-time, representative household, stochastic optimizing model is used to discuss the impact of sustainable development technology on the growth rate of real wealth and the HLM effect. The introduction of additional energy-saving technologies is considered. Our main findings are as follows. First, an innovation in energy-saving technology raises the returns of capital investments and thus increases the investments in capital stocks. The uncertainty in future technology reduces the consumers’ incentive to invest in capital. An increase in energy prices raises the expected return on bonds and reduces capital investments. Finally, these effects on the wealth of a country and the share of consumption are ambiguous and depend on other factors such as portfolio shares and preference parameters.

The displacement effects of energy-saving techniques are stronger, but the instantaneous returns to physical capital are uncertain, and the anticipated (unanticipated) component of technical displacement will increase (reduce) the share of capital and the frequency (possibility) of experiencing a positive growth rate. Thus, the impact of energy-saving techniques on the share of capital and the growth rate depends on the relative magnitudes of its anticipated and unanticipated components, the share of bonds (debtor or creditor), the income and substitution effects, and the representative household’s degree of risk aversion. However, the energy-saving techniques might not affect the conditions under which the HLM effect holds.

The effects of rising energy price are stronger, but the effects on the instantaneous returns of bonds are unclear. The anticipated component of the increase in energy price will increase the share of bonds, but the sum of the portfolio and the unanticipated component of the increase in the energy price will have simultaneous effects on the shares of capital and bonds. Therefore, the impact of an increase in the energy price on the share of bonds and the growth rate depends on the relative magnitudes of its anticipated and unanticipated components, the share of bonds (debtor or creditor), the income and substitution effects, and the representative household’s degree of risk aversion.

For the scarcity of energy, energy price will increase by time, but the degree of uncertainty in the deterioration in TOT and the conditions under which the HLM effect holds differ as a result of the conditions induced by the deterioration in TOT, which is caused by the final good’s price in the foreign country. This result is more complicated than that obtained by Turnovsky [30,31], as Turnovsky considers a greater permanent and temporary energy price and technical displacement shocks than Turnovsky [30,31]. Thus, the results of this paper should be more realistic and should provide more detailed explanations of the impact of energy technological improvement on a small economy.

The economic implications of the above conclusion are as follows: (1) For the growth rate of national wealth, environmental sustainability, and the stability of TOT, governments should improve energy-saving techniques, reduce the uncertainty of the development of energy-saving techniques, and make good use of the financial policies by closely considering the long-term trend and short-term volatility of oil prices. (2) For the growth rate of output and operational sustainability, an enterprise should purchase energy-saving equipment to increase the share of capital and make good use of the financial products related to oil prices. (3) For the growth rate of wealth of the representative household, the household should use the energy-saving equipment and buy the financial products related to energy-saving techniques and oil prices.

Future studies could focus on the impacts of national or regional differences in energy prices, as the International Energy Agency [1] obtained electricity price estimates with significant regional differences, and the highest prices were observed in the European Union and in Japan. Moreover, changes in energy technology (more energy saving) should have regional differences. Therefore, these changes could affect the competitive positions and export potential of nations or industries through their different energy costs. Narayanan and Sahu [2] found that the energy intensities in the aggregate manufacturing, chemical, diversified, food & beverages, machinery, metals & metal products, miscellaneous manufacturing, non-metallic mineral products, textiles, and transport equipment industries from 1990 to 2008 are different in different regions, respectively.

The effects of energy-saving techniques and the energy price increment on macroeconomic variables may differ for small and large economies. Kilian [38] demonstrated that exogenous oil supply shocks had little effect on macroeconomic variables and explained that this was due to the endogenous responses of oil producers elsewhere, U.S. policy makers, etc.

The covariance between energy technical change and an increasing energy price (${\mathrm{Cov}(\mathrm{dP}}_{e}{,\mathrm{dT}}_{e})$) should be positive, as increasing energy prices will induce innovation in capital goods, resulting in less energy intensive production (more energy-saving technologies). Hicks [39] proposed a macroeconomic hypothesis regarding induced innovation that argued for the economization of a factor that has become relatively expensive. There is considerable academic support for the notion that energy-saving technology is a global phenomenon whereby households, firms and governments will develop or use cars, appliances, or industrial equipment that produce more output per unit of energy consumed. Following Schumpeter [40], when energy prices rise, products with energy-saving attributes will spread more rapidly than they would were they to lack such attributes.
